# Specific Therapy for T2 Asthma

**DOI:** 10.3390/jpm12040593

**Published:** 2022-04-07

**Authors:** Diego Bagnasco, Elisa Testino, Stefania Nicola, Laura Melissari, Maria Russo, Rikki Frank Canevari, Luisa Brussino, Giovanni Passalacqua

**Affiliations:** 1 Allergy and Respiratory Diseases, Department of Internal Medicine (DIMI), University of Genoa, 16132 Genoa, Italy; testinoelisa@gmail.com (E.T.); melissarilaura@gmail.com (L.M.); mariarusso28@hotmail.it (M.R.); passalacqua@unige.it (G.P.); 2 IRCCS Policlinico San Martino, 16132 Genoa, Italy; canevari@edu.unige.it; 3 Allergy and Immunology, AO Mauriziano Hospital, University of Turin, 10124 Turin, Italy; stegugu@gmail.com (S.N.); luisa.brussino@unito.it (L.B.); 4 Unit of Otorhinolaryngology-Head and Neck Surgery, University of Genoa, 16132 Genoa, Italy

**Keywords:** asthma, T2 inflammation, monoclonal antibodies, TSLP, tezepelumab, real life, allarmins, severe asthma, biological drugs

## Abstract

Asthma is a disease with high incidence and prevalence, and its severe form accounts for approximately 10% of asthmatics. Over the last decade, the increasing knowledge of the mechanisms underlying the disease allowed the development of biological drugs capable of sufficiently controlling symptoms and reducing the use of systemic steroids. The best-known mechanisms are those pertaining to type 2 inflammation, for which drugs were developed and studied. Those biological treatments affect crucial points of bronchial inflammation. Among the mechanisms explored, there were IgE (Omalizumab), interleukin 5 (Mepolizumab and Reslizumab), interleukin 5 receptor alpha (Benralizumab) and interleukin 4/13 receptor (Dupilumab). Under investigation and expected to be soon commercialized is the monoclonal antibody blocking the thymic stromal lymphopoietin (Tezepelumab). Seemingly under study and promising, are anti-interleukin-33 (itepekimab) and anti-suppressor of tumorigenicity-2 (astegolimab). With this study, we want to provide an overview of these drugs, paying particular attention to their mechanism of action, the main endpoints reached in clinical trials, the main results obtained in real life and some unclear points regarding their usage.

## 1. Background

Asthma is a chronic airways disease, driven by inflammation and characterized by bronchial hyperresponsiveness and reversible expiratory flow limitation [1]. Although the functional aspects of the disease are common to the different types of asthma, the clinical presentation is often heterogeneous among patients. In fact, some types of asthma can be easily manageable, whereas others require more targeted and powerful therapies to provide full symptoms control. Disease heterogeneity is due to different pathophysiological pathways (endotypes), which are clinically expressed in distinct clinical presentations (phenotypes) [2]. The better known inflammatory endotype in asthma, is that called type 2 (T2), because of the main role of type 2 T-helper cell (Th2)-which drive inflammatory responses (interleukin (IL)-4-, IL-5- and IL-13-mediated) [2,3,4], associated to other cells and cytokines such as type 2 Innate Lymphoid Cells (ILC2), IL-33, IL-25 and Thymic stromal lymphopoietin (TSLP) [5,6]. The knowledge of the mechanisms underlying the disease and of the spectrum of cytokines involved in the development of the pathology, made possible to use the latter as a therapeutic target to control patients with difficult-to-control asthma. Essentially, the available therapeutic options, for severe asthmatic people, are monoclonal antibodies (MABs) targeting Immunoglobulin E (IgE), IL-5 or its receptor alpha and IL-4/13 receptor [7]. The principal objective of biological therapies in severe asthma is to ensure disease control in terms of annual exacerbations, use of systemic corticosteroids (CS) either taken daily or in cycles, in case of acute phases of the disease. During the time course of the disease, in type 2 inflammation, other comorbidities could emerge, such as rhinosinusitis with nasal polyposis and or atopic dermatitis. Accordingly, in the case of patients with severe asthma, who are candidates for biological drugs, it is necessary to pay a special attention to all the clinical manifestations of the underlying inflammation, in order to choose the most appropriate drug for the endotypic and phenotypic aspect of patients. The greater knowledge of the mechanisms that regulate inflammation in asthma, in particular those of the type 2 inflammatory pathway, has allowed the development of numerous drugs (some marketed and others currently under study) useful for controlling this pathology. The main properties of these drugs will be dealt with below, both relating to randomized trials and to real life and the knots, still to be solved, of these pathologies. In this review, we will therefore focus on the treatment of patients with a severe form of asthma secondary to type 2 inflammation.

## 2. Identification of T2 Asthma

**Figure 1:**
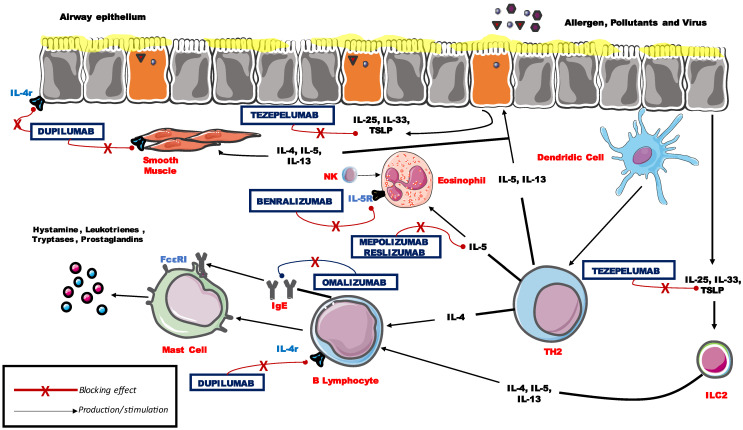
Severe asthma pathway of inflammation and target of MABs.

The increasing knowledge of the pathogenic mechanisms of asthma allowed us to identify several biochemical processes underlying bronchial inflammation. Mainly, two different groups of asthma phenotypes were identified in relation to the inflammatory pathway involved in the immunity cascade. These two groups are named T2 or T2-high (eosinophilic) and non-T2 or T2-low [8]. The T2 pathway is characterized by the involvement of T helper type 2 (Th2) lymphocytes which are able to product, together with ILC2, cytokines or proteins such as IL-4, IL-5, IL-13, while B cells are instead able to produce IgE [3,9,10]. The exposure of airway epithelial cells to specific triggers (i.e., allergens, viruses or irritants in general) prompts to the production and activation of other cytokines, called alarmins (thymic stromal lymphopoietin—TSLP, IL 25 and IL 33), which are able to start a specific inflammatory cascade and therefore result in poor asthma control [11,12]. Allergens can also immediately trigger the bronchoconstriction response, by activating mast cell mediator release, which are, on the other hand, a potential source of T2 cytokines [13]. Eosinophils and mast cells also secrete leukotriene E4 and prostaglandin D2 that stimulate ILC2 cells, leading to a continuous cycle of T2 inflammatory response [13]. IL-5 is the primary regulator of eosinophil proliferation, migration, activation and survival and it also affects the function of mast cells and basophils [14], becoming one of the main pharmacological targets in severe asthma. The other above-mentioned cytokines have a crucial role in inflammation. IL-4 induces Th0 cells to differentiate into Th2 cells and the B cell Ig class switch with the production of IgE [11]. IL-13 and IL-4 induce basolateral secretion of periostin by the epithelial cells, which plays a role in airway remodeling, subepithelial fibrosis, eosinophil recruitment and regulation of mucus production [11]. IgE binds to mast cells and triggers the release of toxic granules [11] (Figure 1). IL-13 stimulates IL-4-induced IgE production by B cells, mucus production, subepithelial fibrosis and Airway hyperresponsiveness (AHR). The degranulation of eosinophils, releasing toxic proteins such as major basic protein, eosinophil peroxidase (EPO), eosinophil cationic protein and eosinophil-derived neurotoxin, has a key role for the hyperresponsiveness and remodeling of the airways.

## 3. Current Therapies in Severe Asthma

Before diagnosing a patient with severe asthma, an accurate therapy titration up to the maximal dosage and a correct adherence to the prescribed therapy are absolutely necessary [11]. Precautions are often sufficient, both in childhood [15] and adulthood, for the control of the disease, patients who are nonetheless still symptomatic, can be considered as severe. Concerning the treatment of these kind of patients, randomized studies on biologics have enrolled patients with a form of asthma, generally defined as severe according to the ATS/ERS guidelines [16]. The main inclusion criteria in pivotal clinical trials follows the guidelines, looking for patients suffering from a form type of asthma treated with maximal therapy following the GINA guidelines (step 4–5) and whose symptoms still remain poorly controlled, requiring supportive steroid therapy, chronic or in cycles (at least 2 per year). Regarding the various drugs, some inclusion and exclusion criteria from the studies were dependent on the biochemical characteristics of the antibody itself, for anti-IgE the IgE count was crucial, as was the eosinophil count for the anti IL-5 or IL-5R and the anti IL-4. Concerning the exclusion criteria, neoplastic patients, patients with a history of smoking greater than 10 pack/y, patients with particular cardiovascular diseases and patients who had already been treated with other biological drugs were not allowed to participate in the randomized trials. Other minor criteria were considered, restricting patient enrollment even further. RCTs criteria drive health authorities to decide protocol for drug administration, shown in Table 1. The selectivity of the criteria for inclusion and exclusion of patients from trials is a well-known problem in the health sector where, generally, the sample examined in clinical studies is not very comparable to those who will then use the drug in “real life” once marketed [17]. The collection of data in real life has allowed us to observe the efficacy and efficiency of biological drugs and how these drugs manage to maintain long-term disease control [18]. 

## 4. Anti-IgE

Omalizumab (OMA) was the first humanized recombinant MAB approved for patients with severe uncontrolled allergic asthma. It binds circulating IgE, inhibiting their binding to the high-affinity receptor on mast cells and basophils [19]. OMA is approved for subcutaneous administration, in severe uncontrolled allergic asthmatic patients [20], aged >6 years, with a positive test (skin test or allergen-specific IgE) to a perennial aeroallergen [21]. More recently, OMA was approved as additional therapy, to intranasal corticosteroids, for adult treatment (>18 years of age) of severe chronic rhinosinusitis with nasal polyposis (CRSwNP) without adequate control of the disease [21,22,23]. There are several OMA clinical studies, with various outcomes such as reduction of exacerbation, symptoms control, quality of life, safety, lung function and reduction of dosage of oral corticosteroids. All the outcomes mentioned above were achieved in regulatory trials, starting from Busse, Humbert and Hanania, who showed a significant reduction of asthma acute episodes, compared to standard of care [19,24,25]. Asthma control, after biological therapy, was evaluated using Total Asthma Symptoms Scores (TASS) with a significant improvement in comparison with placebo (MD −0.16; 95% −0.51 to 0.19). Clinical trials not only evaluated efficacy but also safety, with reassuring data, both in randomized control trials (RCTs) and in real life (RL). Upper respiratory tract infections were the most common reported adverse event and no clinically relevant abnormalities in laboratory tests (including platelets count) were observed [19,26]. Strictly linked to exacerbations was the oral corticosteroids (OCS) sparing effect of OMA, showing a decrease of 45% of OCS use (*p* value 0.002) [27].

### Real-Life Experience with Omalizumab

The favorable results observed in clinical trials were confirmed by numerous real-life investigations carried out worldwide, such as the PROSPERO study including 806 patients taking OMA for 12 months and experiencing a significant reduction in exacerbations (from 3.00 ± 3.28 to a rate of 0.78 ± 1.37; *p* < 0.001), fewer hospitalizations (reduction of 81.9%), and clinically significant improvement in ACT scores when compared with the 12 months before treatment [28]. Finally, the effect of OMA was also evaluated in patients with CRS. In the Proxima study, patients were divided into with and without this comorbidity, demonstrating that the presence of CRSwNP did not negatively influence the response to OMA treatment in terms of improvement in asthma control and lung function or in reduction of annual asthma exacerbation rate [29]. In addition, OMA was approved in pregnancy. A study compared the prevalence of congenital anomalies in asthmatic patients, treated with OMA during pregnancy, and a cohort of non-treated patient, reaching the conclusion that no difference between two groups was observed [30]. 

## 5. Anti-IL-5 and IL-5Rα blockers

Eosinophils are one of the best-known targets of biological drugs in severe asthma; the well-known role of IL-5 on these cell’s maturation, development and growth led to the choice of this cytokine, or its receptor, as a pharmacological target [31,32]. Currently, there are three drugs acting on IL-5 or its receptor, mepolizumab, reslizumab and benralizumab.

**Mepolizumab** (MEP) is a humanized monoclonal antibody, belonging to the class of IgG1κ, able to block the interaction between the α-subunit and IL-5R on the eosinophil cell surface. The inhibiting action of the drug induces an inactivation of eosinophil maturation, activation and growth [7,33]. MEP is given at the dose of 100 mg subcutaneously every 4 weeks, in patients > 12 years old, with severe asthma and a number of eosinophils greater than 300 cells/µL in the year previous to the administration and at least 150 cells/µL at the moment of first dose. 

Similarly to the previously mentioned drugs, MEP trials had, as their primary endpoint, the efficacy of the drug in reducing exacerbations and the role of the drug in OCS sparing. After questionable results, in a patient sample pool that was selected [34], the registration trials showed a relevant effect both in the reduction of exacerbations and in OCS sparing [35,36,37,38] (Table 2). Several studies are also available in real life, contributing to our knowledge further information on the drug [39,40,41,42,43,44]. The extension studies of main clinical trials, COSMOS [45], COLUMBA [46] and COSMEX [47], and others in real life, have also made it possible to highlight how the safety profile, even in the long term, is very reassuring, with confirmation that adverse events are very rare and mild. 

### 5.1. MEP in Real Life

The real-life experience of MEP highlighted interesting aspects for which, at the moment, no in-depth analysis has been carried out in the RCTs. Pharmacoeconomic surveys highlighted how virtuous the drug is and capable of self-financing part of the annual expenditure, even if only by reducing the indirect costs due to absenteeism from work and not even taking into consideration the amount that could be saved in terms of reduction of comorbidities from chronic steroid therapy [56]. In addition, to these data, the experience in real life has also made it possible to highlight the characteristics of subjects, definable as “super responders”, capable of having a greater response to the drug, found in patients with nasal polyposis, a lower BMI and a lower maintenance prednisolone requirement at baseline [57]. 

**Reslizumab** (RES) is an MA, belonging to the class of IgG4κ, which, as well as MEP, prevents the binding of IL-5 to its receptor alpha [33]. Despite that RES was approved by the FDA and EMA [7] for the treatment of patients >18 years with a history of severe eosinophilic asthma, in Italy and several other European countries, it was not marketed. Unlike MEP, RES can only be used intravenously at variable doses, depending on weight, every 4 weeks in patients with more than 400 eosinophils/µL [58]. The cut-off point of 400 cells was established based on a trial where it was found that patients, with an eosinophilic count greater than 400, were able to respond better to the drug, significantly reducing exacerbations and the need of secondary therapies and improving respiratory function [48]. Another study by Castro confirmed that RES could reduce exacerbations at a rate of 32% (*p* < 0.0001) versus placebo [49]. Long-term real-life studies provided additional data about efficacy and safety [59,60]. 

### 5.2. RES in Real Life

As for MEP, RES demonstrated efficacy and efficiency in real life evaluations, also showing a good short- and long-term safety profile [52].

**Benralizumab** (BEN) is a humanized, afucosylated monoclonal antibody able to bind the alpha subunit of interleukin-5R; it is also an anti-eosinophilic drug, but with a different mechanism than those described above. The binding of eosinophil’s alpha subunit can generate apoptosis through antibody-dependent, cell mediated cytotoxicity. Although at the blood level, the eosinophil count, after administration of the drug, falls to zero, bronchoscopic studies demonstrate that eosinophilia reduces more than 90% of airway mucosa and sputum [52]. 

The efficacy of BEN was confirmed in clinical trials and real-life settings. The most important trials, CALIMA and SIROCCO, demonstrated the role of the drug (30 mg subcutaneously first every 4 w for 3 months and then every 8 w) in reducing exacerbations, improving Forced Expiratory Volume in the 1st second (FEV1) and reducing the use of OCS in treated patients [50,51]. 

### 5.3. BEN in Real Life

As for the other drugs, and also for BEN and RL, evidence of their efficacy as described in clinical trials is confirmed [61]. Similarly to what happened for OMA, in the abovementioned sub analysis of Proxima, a sub analysis of patients affected by CRS in RL setting was performed for BEN, confirming the efficacy of the drug both in patients with and without rhinosinusitis and observing a non-significant but evident trend of improved response in asthmatic patients with CRS when compared to the one without [62]. Once more, the real-life studies allowed us to define the characteristics of those patients that are definable as super responders, which once again turned out to be those with rhinosinusitis and with a greater use of OCS [63].

## 6. Anti-IL-4r

Dupilumab (DUP) is a fully human monoclonal antibody binding IL-4Rα that, by being common to both IL-4 and IL-13, is capable of inhibiting the signaling of both [6,11]. DUP was approved in asthmatic subjects with underlying type 2 inflammation, aged ≥12 years, not controlled by maximal therapy. Therapeutic dosing of DUP (s.c.), administered with an initial loading dose of 600 mg (two 300 mg injections) and continued with 300 mg/2 weeks (recommended only for patients with OCS-dependent asthma or with comorbidities), or 400 mg (two 200 mg injections) and followed by 200 mg/2 weeks, was investigated following findings from clinical trials. Data on asthma control improvement, in terms of exacerbations and FEV1, were found in a phase II study with variable doses (200 or 300 mg every 2 or 4 weeks) where patients with moderate-to-severe asthma were treated [52], showing improvement in the target outcomes in all patients, although the best results were seen in those with ≥300 eosinophils/µL. The clinical outcomes that were evaluated in the trials were the reduction in the frequency of exacerbations, the ability to reduce systemic steroid therapy and the improvements in lung function. They were found to be greater in asthmatics with a baseline blood eosinophil count greater than 150 cells/microliter or fractional exhaled nitric oxide FeNO values greater than 25 parts per billion (ppb) [64,65,66]. Among the adverse events of DUP, hypereosinophilia was the most common (with blood values greater than 1500 per microliter) in a variable percentage, between 4% and 25% of patients; this increase seems likely to persist for more than 6 months in 14% of these patients [67]. Although, in most cases, DUP-induced hypereosinophilia is asymptomatic, a few cases of eosinophilic granulomatosis with polyangiitis and eosinophilic pneumonia have been reported [67,68,69]. 

### DUP in Real Life

Although there are few studies, considering the brief commercialization of the DUP, in this case, the real-life studies have also allowed us to confirm the effectiveness of the drug. The authors of a multicenter work, lasting for 1 year, also proved the drug’s safety in patients who have experienced hypereosinophilia, with values higher than 1500 cells/uL (25% of the sample) [67].

## 7. Blocking Antibodies Targeting Epithelial Cell-Derived Cytokines

As previously mentioned, the role of cytokines produced by epithelial tissue has been found to be central in the development of the disease and together with IL-4, IL-5 and Il-13, also IL-25, IL-33 and TSLP were identified as possible therapeutic targets in asthma. 

In severe asthmatic patients, RCTs evaluated the efficacy of anti-TSLP (tezepelumab), anti IL-33 human monoclonal antibody (itepekimab) and an anti-IL-33r (also known as suppressor of tumorigenicity 2 [ST2]) (astegolimab). 

RCTs with Tezepelumab demonstrated a reduction of annualized asthma exacerbation rate by 56%, in patients with more than 300 eosinophils/µL, treated with 210 mg of drug administered subcutaneously every 4 weeks and by 41% in the one with less than 300 cells/µL [53]. In clinical trials, the efficacy of Tezepelumab has been demonstrated to also improve lung function, asthma control and in decreasing FeNO and blood eosinophils level and in reducing hyperresponsiveness to mannitol in treated patients [54,55]. Due to the results of clinical trials, Tezepelumab has been approved for severe asthma in US in patients elder than 12 y and it is the only biological drug approved without phenotype limits. It is also currently being validated in Europe and Japan, and it is in an advanced phase of study for the treatment of COPD, CRSwNP and chronic spontaneous urticaria [70].

Anti IL-33 targeted drugs, Itepekimab, was evaluated at a dose of 300 mg, with a biweekly subcutaneous administration, in moderate-severe asthmatic in therapy with ICS and LABA [66]. 

Astegolimab, administered subcutaneously every 4 weeks at the dose of 70 mg or 490 mg (but not 210 mg), is able to decrease exacerbations, in comparison to placebo administration, in a phase 2b RCT involving patients with severe asthma, also including those with low eosinophil counts [71].

## 8. Future Perspective and Unmet Needs

That described so far clearly demonstrates the efficacy of biologic drugs in the treatment of patients with severe type 2 asthma (Table 1). The mechanisms on which the various antibodies act can be different, sometimes having different objectives, sometimes having the common objective of reducing eosinophilic inflammation. In daily clinical practice, the problem of choosing the most appropriate drug for the patient is arising. The choice of one drug rather than another is currently guided by the presence of some systemic “markers”, such as IgE, eosinophils or volatiles such as FeNO and integrated by the clinical features, which are essential to better assess the patient. Among the “macroscopic” markers that can guide the clinician’s choice of a drug rather than the other, there may also be comorbidities that accompany the asthma of the patient. Nasal polyposis, for example, is often associated with asthma [72,73,74], frequently driven by a type 2 inflammation [75,76,77,78]; it has been found to be responsive to some of the drugs described above [79,80,81,82] and the presence or absence of this manifestation may lead to the choice of a specific drug. An additional point of insight regarding biological drugs is the duration of their use [83]. It is well known that the administration of monoclonal antibodies does not affect the natural history of the disease, but in fact aims to modulate an inflammatory mechanism, exaggeratedly or not physiologically expressed. Precisely for this reason, it is expected that when the treatment is suspended, this mechanism may return to being pathogenic and favoring poor control of the disease. Regarding the discontinuation of the drug, there is not a common consensus. Some authors propose the discontinuing of the treatment in particular cases, in which patients can fall within a set of parameters that define them as controlled [84], while other authors suggest grater caution. Studies performed on the oldest drugs on the market (OMA and MEP), and therefore on patients who may have been taking them for a long time, suggest that caution be applied. Over the past few years, evidence in real-life studies and in RCTs have shown that the suspension of drugs, only in a set of patients, allows us to maintain control of the disease, while instead, others are forced to reintroduce OCS or the suspended drug, in order to reduce the new onset of symptoms [85,86]. In the XPORT study, 88 patients suspended OMA and as many continued with therapy, observing a greater exacerbation rate in the group of discontinuation (52.3 vs. 33.0%) [87]. Very similar results were also shown in the COMET study, where discontinuation was attempted, in a double-blind mode, in patients treated with MEP for about 5 years, observing that those who stopped MEP had more exacerbations compared to those who continued the therapy (61% versus 47%, respectively) [88]. These and other observations call for greater caution and the need for further studies before defining a category of patients whose characteristics are worthy of discontinuing the drugs that are currently in use for severe asthma. A further aspect of great interest is the use of multiple monoclonal antibodies in selected patients for whom one drug has not been found to be sufficient. In the literature, only a few anecdotal cases regarding this point are found. Generally, the single therapy is effective in controlling symptoms in asthmatic patients; however, in some more complex cases, the combination of several drugs could be desired in order to obtain a more ubiquitous regulation of the inflammatory pattern [89,90,91,92,93]. The forthcoming marketing of drugs with actions further upstream in the inflammatory cascade of asthma could partly solve this problem. This would therefore provide a product that can act not only on a cytokine or a cell, but also on a more complex system that, in the cascade, modulates a greater series of mediators.

## 9. Conclusions

Monoclonal antibodies have been found to be effective drugs in adjunctive therapy for uncontrolled severe asthma in individuals with type 2 inflammation.

Indeed, the drugs that are currently on the market showed a good efficacy and safety in controlling the exacerbations, reducing the use of systemic corticosteroids and improving the quality of life of regularly treated patients. In the near future, other drugs will be available in order to offer a therapeutic alternative, and also in patients for whom the current therapies are not sufficient and, in some cases, even for those who are found to have an endotype more distant from type 2 inflammation. For these patients, the therapeutic choice remains limited. With the continuation of this research, it will also be possible to provide answers to questions that are still unanswered, such as the duration of the therapies and what the process of choice of biological drugs in patients who have the characteristics to respond to multiple drugs can be.

## Figures and Tables

**Table 1:** Characteristics about prescribing criteria of MABs in severe asthma.

Drug	Administration Criteria
Omalizumab	6–12 y Severe asthma IgE mediated In vitro or cutaneous positivity for perennial allergen Frequent exacerbations/OCS dependent High dose of ICS and second controller LABA 12 y All the previous mentioned FEV1 < 80% Dosage to be defined according to weight and total IgE count
Mepolizumab	>6 y Severe uncontrolled asthma Eosinophils > 300 cells/µL in previous 12 months and >150 cells/µL in the moment of administration, without systemic steroid treatment ≥2 exacerbations, requiring OCS in previous 12 months or chronic OCS therapy for at least 6 months in the last year
Reslizumab	>18 y Severe uncontrolled asthma Eosinophils > 400 cells/µL without systemic steroid treatment or, in long term OCS treated with eosinophil count <400 cells/µL; a pre-OCS eosinophil level should be used to confirm eosinophilic phenotype. ≥1 exacerbation, requiring OCS in previous 12 months
Benralizumab	>18 y Asthma therapy according to Step 4–5 GINA Eosinophils > 300 cells/µL without systemic steroid treatment ≥2 exacerbations, requiring OCS in previous 12 months or chronic OCS therapy in the last year
Dupilumab	>12 y Severe asthma with T2 inflammation Asthma therapy according to Step 4–5 GINA Eosinophils ≥ 150 cells/µL or FeNO > 25 ppb ≥2 exacerbations, requiring OCS in previous 12 months or requiring hospitalization, or chronic OCS therapy for at least 6 months in the last year

**Table 2:** Main characteristics of MABs in severe asthma.

Drug	Dose and Route of Administration	Patient Characteristics	Efficacy	Main Side Effects	Other Diseases Approvation
**Anti IgE**					
Omalizumab [24,25]	Subcutaneous (SC) administration. Prefilled syringe 75 to 600 mg every 2 to 4 wk according to: Serum total IgE level (IU/mL), measured before the start of treatment; Body weight (kg).	Moderate to severe persistent asthma in adults and pediatric patients **6 years** of age and older with a positive skin test or in vitro reactivity to a **perennial aeroallergen** and symptoms that are inadequately controlled with inhaled corticosteroids and second controller.	-Reduced exacerbations; -Reduced symptoms; -Small effect on FEV1; -Improved quality of life.	Injection side reaction, fever, arthralgia, fatigue, bone fracture, nausea, abdominal pain, pruritus, dermatitis, earache, hypereosinophilic conditions (e.g., EGPA), abrupt discontinuation of oral glucocorticoids; black-box warning for anaphylaxis.	Nasal polyps; Chronic spontaneous urticaria (CSU).
**Anti IL-5**					
Mepolizumab [35,36,37,38]	Subcutaneous (SC) administration. Prefilled syringe Autoinjector pen Adults and adolescents: 100 mg every 4 wk. Children, ages 6–11 yr: 40 mg every 4 wk.	Add-on maintenance treatment of adult and pediatric patients aged **6 years** and older with severe asthma and with an **eosinophilic phenotype (>300 cell/µL previous 12 m and > 150 at the moment of first administration)**.	-Reduced exacerbations; -Reduced symptoms; -Small or moderate effect on FEV1; -Reduction or withdrawal of oral glucocorticoids if blood eosinophils > 150/µL; -Improved quality of life.	Headache, injection site reaction, back pain, arthralgia, fatigue, helminthic infections, hypersensitivity reactions, abrupt discontinuation of oral glucocorticoids, Herpes Zoster infections (rare).	Chronic rhinosinusitis with nasal polyps (CRSwNP) ^¥^; Eosinophilic granulomatosis with polyangiitis (EGPA) ^¥^; Hypereosinophilic syndrome (HES) ^¥^.
Reslizumab [48,49]	Intravenous infusion only. Recommended dosage regimen is 3 mg/kg once every 4 weeks by intravenous infusion over 20–50 min.	Add-on maintenance treatment of patients with severe asthma **aged 18** years and older and with an **eosinophilic phenotype (>400 cell/µL)**.	-Reduced exacerbations; -Reduced symptoms; -Small or moderate effect on FEV1; -Improved quality of life.	Oropharyngeal pain, helminthic infections, abrupt discontinuation of oral glucocorticoids, black-box warning for anaphylaxis.	
**Anti IL-5R**					
Benralizumab [50,51]	Subcutaneous injection. Prefilled syringe Autoinjector pen Recommended dose is 30 mg every 4 weeks for the first 3 doses, followed by once every 8 weeks thereafter.	Add-on maintenance treatment of patients with severe asthma **aged 12** years and older and with an **eosinophilic phenotype (>300 cell/µL)**.	-Exacerbations; -Reduced symptoms; -Small or moderate effect on FEV1; -Decrease or withdrawal of oral glucocorticoids if blood eosinophils > 150/µL; -Improved quality of life.	Helminthic infections, hypersensitivity reactions, abrupt discontinuation of oral glucocorticoids.	
**Anti IL-4R**					
Dupilumab [52]	Subcutaneous injection. Prefilled syringe Autoinjector pen (in CRSwNP) -Initial loading dose 400 mg (two 200 mg injections), subsequent dose 200 mg every 2 weeks. **OR** -Initial loading dose 600 mg (two 300 mg injections), subsequent dose 300 mg every 2 weeks. (For patients with OCS-dependent asthma or comorbidities *) **OR** -Dosage in Pediatric Patients 6 to 11 Years of Age (§).	Add-on maintenance treatment of adult and pediatric patients **aged 12 years** and older with moderate-to-severe asthma characterized by an **eosinophilic phenotype or with oral corticosteroid dependent asthma (eosinophils ≥ 150 cell/µL; FeNO > 25 ppb)**.	-Reduced exacerbations; -Reduced symptoms; -Improved lung function; -Decrease or withdrawal of oral glucocorticoids; -Irrespective of blood eosinophil count at baseline; -Improved quality of life.	Injection site reactions, oropharyngeal pain, eosinophilia, helminthic infections, hypersensitivity reactions, abrupt discontinuation of oral glucocorticoids, hypereosinophilic conditions (e.g., EGPA), conjunctivitis.	Atopic Dermatitis; Chronic Rhinosinusitis with Nasal Polyposis (CRSwNP).
**Anti TSLP**					
Tezepelumab ^µ^ [53,54,55]	Administer by subcutaneous injection. Recommended dosage is 210 mg administered once every 4 weeks.	Add-on maintenance treatment of adult and pediatric patients **aged 12 years** and older with **severe asthma**.	-Reduced exacerbations; -Reduced symptoms; -Improved lung function; -Improved quality of life.	Pharyngitis, arthralgia, back pain, hypersensitivity reactions, helminthic infections, abrupt discontinuation of oral glucocorticoids.	

^µ^ Not yet marketed. * Dosage for patients with oral corticosteroid-dependent asthma or with co-morbid moderate-to-severe atopic dermatitis or adults with co-morbid chronic rhinosinusitis with nasal polyposis. § Where approved, for pediatric patients, ages 6 to 11 yr, with a body weight of 15 kg to less than 30 kg, the recommended dose of dupilumab is 100 mg every 2 wk or 300 mg every 4 wk; for children with a body weight of 30 kg or more, the dose is 200 mg every 2 wk. For pediatric patients 6 to 11 years old with asthma and co-morbid moderate-to-severe atopic dermatitis: −15 to less than 30 kg: initial dose 600 mg (two 300 mg injections), subsequent dose 300 mg every 4 weeks (Q4W). −30 to less than 60 kg: initial dose 400 mg (two 200 mg injections), 200 mg every other week (Q2W). −60 kg or more: 600 mg (two 300 mg injections) 300 mg every other week (Q2W). ^¥^ EMA and FDA approved.

## Data Availability

Not applicable.
